# Progress in Medicine

**Published:** 1898-01-08

**Authors:** 


### Progress in Medicine.

### FEVERS.

(*Continued from page* 243.)

**Beri-beri** has been the subject of numerous pathological investigations by Pekelharing, Winkler, and others. W. K. Hunter[15] has made careful cultivations from the blood of two patients, and confirms in every particular those observers who regard as the specific agent a certain white staphylococcus. He found this micrococcus in the blood on every examination, and demonstrated a micrococcus in cultures made from it. The injection of these cultures into rabbits was followed by signs of paralysis, and peripheral neuritis; the special lesion of beri-beri was found to exist in these rabbits after death, and the micrococcus itself was found to exist in their bodies. Certain bacilli were also found of various kinds, but none regularly, and none of them appear to be of importance. The author, while confessing that this staphylococcus is not unlike the S. pyogenes albus, shows that its powers are totally different, though whether it be originally developed in any way from the allied organism is unknown.

**Yellow Fever.**—Besides Sanarelli, another claimant has appeared for the honour of demonstrating the cause of this disease. Havelburg[16] brings forward a small, straight bacillus, a half or a quarter the size of Sternberg's. Both are able to exist without free oxygen, are frequently found in pairs, and both ferment sugar. Havelburg's form is found in the stomach, curdles milk, and produces indol but no toxin. Sanarelli's lives in the tissues and blood, is not affected by dessication, and can therefore be carried by the air, and finally produces a virulent poison, very little of which is needed to produce the worst type of the disease. Moreover, it favours the growth of other microbes, and in cultures its own growth is greatly facilitated by the presence of moulds. Still, if it is, as Sanarelli claims, pathogenic for the lower animals, it is not clear why the animals tested by the Commission in Cuba some years ago all escaped. Small quantities of the filtered and sterilised cultures produced in human beings all the symptoms of the disease, so that there seems little chance of using protective injections of this very powerful toxin.

**The Plague.**—Wyssokowitch and Jobobat[17] consider that the mortality might be reduced to half by the use of Yersin's serum with careful dosage, and in the really early stages of the disease. The protective action in their experiments on monkeys did not appear to last more than a fortnight, and they consider that the healthy skin can be traversed by the bacillus, which also enters through the lungs and mouth. Haffkine[18] claims that in an outbreak among prisoners only two non-fatal cases occurred out of 148 persons inoculated with sterilised cultures of the bacillus, while six fatal and six mild cases occurred in the 173 non-inoculated. He went on to treat no less than 11,000 persons with this fluid with a large diminution of the death-rate. Lustig and Galeotti[19] prepared a dried vaccine which in measured quantities could be injected into animals, causing them to form a curative and protective serum in about two weeks; moreover, it would seem possible to use the vaccine itself for human protection, as it is quite harmless in small doses. The bacillus itself appears from Abel's researches[20] to be very resistant, living in cloth fabrics for a month, and persisting in the blood and urine of patients after convalescence, and even in the water of wells. Carbolic solutions of less strength than one in 20 have no effect upon it. At Mandvi there were 721 persons vaccinated by Yersin's serum, and N. Dempster Mason found some time after that not one of them had been attacked,[21] but he complains that the period of immunity is so short that the necessary frequent vaccinations are impossible. He used the serum also for 30 patients as a means of cure, and found the mortality reduced 23 per cent. The serum requires 24 hours to act, and is therefore useless in the severest attacks.

15 Lancet, July 31. 16 Amer. M. S. Bullet., Aug. 25. 17 Lancet, July 24. 18 B. Med. Jour., June 12 19 B. Med. Jour., Ap, 24. 20 B. Med. Jour., June 12. 21 Birmingham Med. Rec., Sept.

